# The role of zinc in the stability of the marginally stable IscU scaffold protein

**DOI:** 10.1002/pro.2501

**Published:** 2014-06-11

**Authors:** Clara Iannuzzi, Miquel Adrover, Rita Puglisi, Robert Yan, Piero Andrea Temussi, Annalisa Pastore

**Affiliations:** 1 MRC National Institute for Medical ResearchThe Ridgeway, London, NW7 1AA, United Kingdom; 2 Department of Biochemistry, Biophysics and General Pathology, Seconda Universita' di Napoli80138, Naples, Italy; 3 Institut Universitari d'Investigació en Ciències de la Salut (IUNICS). Departament de Química, Universitat de les Illes BalearsCtra. Valldemossa km 7.5, E-07122, Palma de Mallorca, Spain; 4 Dipartimento di Chimica, Universita' di Napoli Federico IIVia Cinthia, I-80126, Napoli, Italy; 5 Department of Clinical Neurosciences, King's College LondonLondon SE5, United Kingdom

**Keywords:** iron–sulfur clusters, metal coordination, metalloprotein, protein stability, zinc binding protein

## Abstract

Understanding the factors that determine protein stability is interesting because it directly reflects the evolutionary pressure coming from function and environment. Here, we have combined experimental and computational methods to study the stability of IscU, a bacterial scaffold protein highly conserved in most organisms and an essential component of the iron–sulfur cluster biogenesis pathway. We demonstrate that the effect of zinc and its consequence strongly depend on the sample history. IscU is a marginally stable protein at low ionic strength to the point that undergoes cold denaturation at around −8°C with a corresponding dramatic decrease of enthalpy, which is consistent with the fluxional nature of the protein. Presence of constitutively bound zinc appreciably stabilizes the IscU fold, whereas it may cause protein aggregation when zinc is added back posthumously. We discuss how zinc coordination can be achieved by different side chains spatially available and all competent for tetrahedral coordination. The individual absence of some of these residues can be largely compensated by small local rearrangements of the others. We discuss the potential importance of our findings *in vitro* for the function *in vivo* of the protein.

## Introduction

IscU is a small (120 amino acids) bacterial protein with close eukaryotic orthologues. Its cellular role is that of transiently hosting iron–sulfur cluster groups.^1^–^4^ These are essential prosthetic groups exploited by the cell because of their favorable redox potential.^5^,^6^ In most organisms, IscU works in cooperation with IscS (Nfs1 in eukaryotes), a desulfurase that converts cysteine into alanine and transfers a highly reactive persulfide to IscU to allow cluster formation.^7^ The interaction between IscU and IscS is weak, with the complex of the *Escherichia coli* proteins having micromolar dissociation constant.^8^

The structures of several IscU orthologues have been described.^9^–^11^ Although sometimes referred to as a dimer, isolated IscU has consistently been reported to be a monomer in solution.^6^,^12^ IscU is also an obliged monomer when bound to IscS since the binding sites on the IscS dimer are distant and on opposite surfaces.^13^–^15^ In a crystal structure of the isolated protein, IscU is a trimer with the cluster bound only on one of the subunits.^11^ This is, however, thought to be a crystallization artifact.

Coordination of the cluster is suggested to occur through three highly conserved cysteines (Cys37, Cys63, and Cys106) present on the tip of the approximately ellipsoidal shape of IscU. Spatially close to the cysteines are also two residues that can act as potential ligands, an aspartate and a histidine (Asp39 and His105 according to the *E. coli* numbering), although their precise role in cluster coordination remains unclear. An IscU mutant where the well-conserved Asp39 is substituted by an alanine is known to lead to more stable iron–sulfur cluster coordination.^16^

In most of the PDB entries, IscU consists of two helices packing against a contiguous three-stranded β-sheet.^12^ The first helix is flexible and largely unstructured in solution but folds back and packs against the rest of the globular domain in the X-ray structures both of isolated IscU and in the complexes with IscS.^13^,^14^ In some of the crystal structures, a zinc metal ion is observed at the site, which hosts the cluster in the cluster-loaded protein.^9^,^10^,^17^,^18^ The presence of zinc is also identified in structures solved by nuclear magnetic resonance (NMR).^19^ This cation is thought to stabilize the fold.^20^ For this reason, Zn^2+^ is often introduced in the culture medium to facilitate IscU overexpression in the stably folded form.^21^

In the absence of zinc, IscU was shown to be present as an equilibrium between two distinct forms, one of which being fully structured (S), the other being partially disordered (D).^20^ It was also suggested that the flexibility of the IscU fold might be an inherent property of the protein, necessary for its function. No conclusive evidence is, however, currently available to support this hypothesis. A recent elegant report showed, however, that the presence of zinc is necessary for the structural and catalytic competence of the close analogue SufU protein of *Bacillus subtilis*.^22^

As a way to better understand the role of zinc, we present here a study on how this cation and other parameters such as ionic strength and pH affect fold stability and the functional role of IscU. We identified the residues involved in zinc coordination and tested whether and how the presence of the cation influences the interaction with its main partner, the desulfurase IscS, and the capacity to accept the cluster. Our results show that zinc has a strong affinity for IscU and that its presence, far from interfering with iron–sulfur cluster formation, enhances its efficiency. We discuss how zinc could contribute to the cellular function of the protein.

## Results

### It is possible to distinguish the state of Zn^2^^+^on IscU through its stability profile

**Figure 1:**
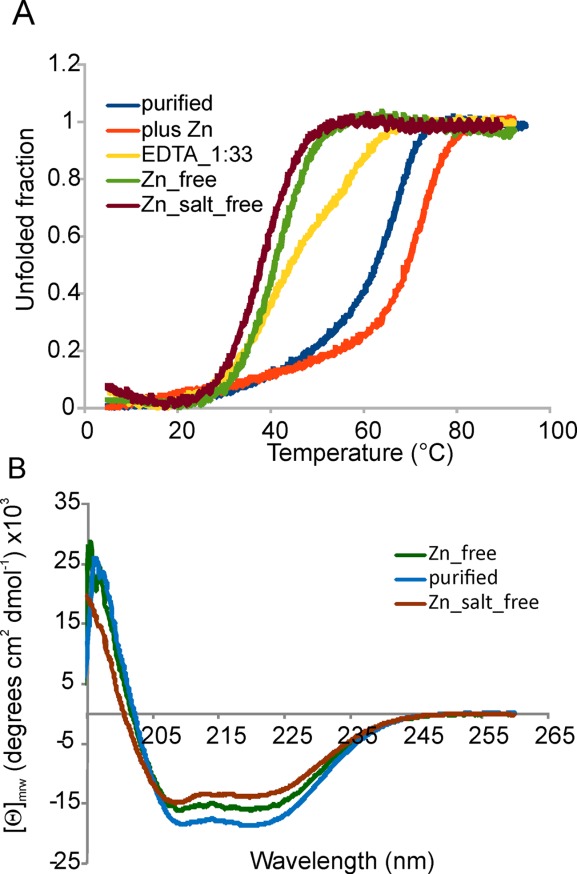
CD properties of IscU_wt as purified, Zn^2+^ depleted, Zn^2+^ depleted and desalted, and after adding extra Zn^2+^ to the solution. (A) Comparison of the temperature scans following the wavelength at 222 nm. (B) Comparison of the far-UV spectra showing a lower helical content in the absence of Zn^2+^ and at low ionic strength.

To characterize the effects of zinc on the stability of IscU, we recorded thermal scans monitored by circular dichroism (CD) spectroscopy following the signal at 222 nm wavelength [Fig. 1(A)]. We determined the unfolding profile of IscU as freshly produced (IscU_wt) by *E. coli* overexpression (that is in the natural host of this protein). No additional Zn^2+^ was added to the culture medium^23^ to ensure that the sample would not be artificially enriched with this cation.

The resulting midpoint temperature of the unfolding transition (*T*_m_) at pH 8 (in 20 m*M* phosphate buffer, 150 m*M* NaCl, and 0.5 m*M* tris(2-carboxyethyl)phosphine (TCEP)) is 65°C (Table I). When the sample was pretreated with ethylenediaminetetraacetic acid (EDTA) to remove possible divalent cations (including Zn^2+^), we observed a substantial decrease of the *T*_m_ in agreement with the stabilizing effect of the cation. However, when we used an excess of EDTA (1:33 IscU:EDTA molar ratio), we observed an unfolding profile that is not that of a two-state unfolding and has two transitions, one around 35°C, the other at about 59°C [Fig. 1(A), yellow curve]. A single highly cooperative transition was observed only when using a large excess of EDTA (i.e., 25 m*M* that corresponds to a 1:500 protein:EDTA molar ratio) indicating that binding is tight (below the *K*_d_ of EDTA for zinc, i.e., 10^−12^ *M*).

**Table I:** Thermodynamic Parameters for IscU_wt as Obtained From the CD Data

	*T*_m_ (°C)	Δ*H*_m_ (kcal/mol)	Δ*C*p (kcal/(mol K)	Δ*S*_m_ (Kcal/mol K)	T_s_ (°C)
As purified	65.0	52.0	1.74	0.15	35
Zn^2+^_free	41.8	49.1	2.08	0.16	19
Added Zn^2+^	72.6	69.6	1.59	0.20	29
Zn^2+^/Salt free	39.1	47.7	2.15	0.15	15
Fe^2+^^a^	67.5	53.8	1.70	0.16	35
Fe^3+^^a^	68.0	54.0	1.68	0.16	36

The midpoint of the thermal transition *T*_m_ and the temperature of maximal stability T_s_ are expressed in Celsius instead of Kelvin to make it easier to appreciate the values. The thermodynamic parameters were calculated as described in the Experimental Procedures.

a The values refer to experiments in which 150 µ*M* Fe(II or III) was added under anaerobic and aerobic conditions, respectively, to as purified IscU.

For comparison, the temperature scan on the Zn^2+^ free protein revealed a dramatic destabilization, with a *T*_m_ of 41°C. When an excess of Zn^2+^ (1:3 molar ratio) was added to the EDTA pretreated sample to saturate the protein, we observed a substantial stabilization of the protein which unfolds with a *T*_m_ of 73°C.

Finally, we added Fe^2+^ under strict anaerobic conditions to the EDTA pretreated IscU_wt to test whether zinc could be replaced by iron, which is somewhat a more obvious partner of the protein. This cation had only a minor effect (i.e., a 4°C increase of the melting point, data not shown) suggesting that, when not in the cluster, iron binds less tightly than Zn^2+^. This is in agreement with our previous NMR studies, which did not support direct binding of the cation to as purified IscU_wt.^24^

Taken together, these data indicate that constitutively incorporated Zn^2+^ has a strong affinity for IscU according to what observed for SufU^22^ requiring a huge excess of EDTA to strip away the cation and provide a reliable way to test whether the protein is sufficiently Zn^2+^ depleted to show a two-state unfolding behavior.

### IscU_wt undergoes cold denaturation under low salt concentrations

Next, we tested the effect of ionic strength on IscU_wt stability. EDTA pretreated IscU_wt was eluted through a desalting column to remove NaCl. Comparison of the overall far-UV CD spectrum of IscU_wt as purified, Zn^2+^ depleted and Zn^2+^/salt depleted indicates a significant loss of secondary structure (ca. 25%) when no salt is present [Fig. 1(B)]. The *T*_m_ value decreases further as compared to the Zn^2+^ free form (39.1°C) indicating that the ionic strength has a further stabilizing effect on IscU_wt in addition to Zn^2+^ (Table I).

As previously described [20], in addition to the heat-promoted transition, we observed a curvature of the signal at low temperature, which is indicative of cold denaturation^25^ and would lead to a second transition around −8°C [Fig. 1(A), green and brown curves]. This behavior allows the calculation of the whole stability curve (i.e., the plot Δ*G* of unfolding vs. temperature), which provides an accurate estimate of the thermodynamic parameters (Table I). For samples containing zinc, which show no cold denaturation, it is necessary to use predicted values of Δ*C*_p_ to calculate thermodynamic parameters. The value of Δ*C*_p_ calculated for the folded part (less than 100 residues) of IscU is of the order of 1.4 kcal/mol/K.^26^ Using this figure yields a significant drop of Δ*H*, that is, from about 70 kcal/mol for the zinc loaded protein to about 48 kcal/mol for the salt free protein. The decrease of Δ*H* and Δ*S* at the transition point is consistent with a fluxional nature of the protein in the absence of salt.

IscU is thus a protein only marginally stable in the absence of stabilizing partners.

### How is Zn^2^^+^ coordinated?

**Figure 2:**
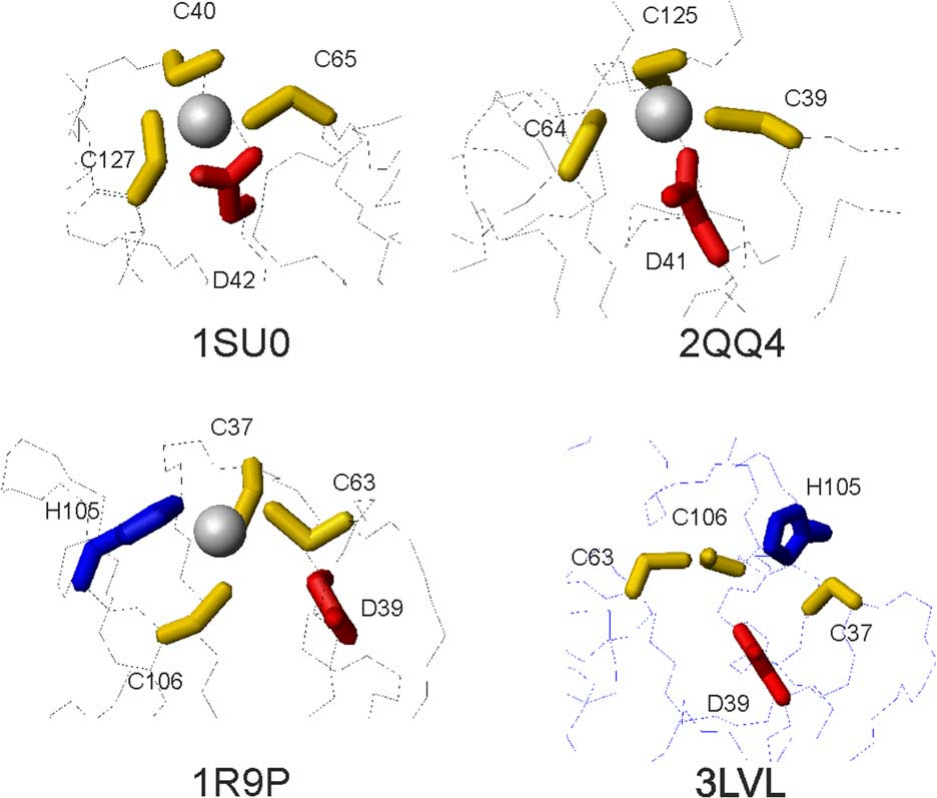
Comparison of the zinc coordinating region in the available PDB entries. 1SU0, 2QQ4, 1R9P, and 3LVL are the structures of IscU from *S. pyogenes*,^18^ *T. thermophilus HB8*, *H. influenzae*,^10^ and *E. coli*,^14^ respectively. 1SU0, 2QQ4, and 3LVL are crystal structures solved at 2.30, 1.85, and 3.00 Å resolution. Only 1SU0 contains explicitly zinc coordinates. 1R9P is an NMR structure, which Zn^2+^ was modeled in.

To characterize zinc coordination, we inspected in detail all the available IscU-related structures (Fig. 2). Only two of them, obtained by X-ray crystallography at a 2.30 Å resolution, namely IscU from *Streptococcus pyogenes* (pdb code: 1SU0) contains experimentally determined Zn^2+^ coordinates.^9^ In this structure, the cation is surrounded by three conserved cysteines (Cys40, 65, and 127 that correspond to Cys37, 63, and 106 in *E. coli* residue numbering) that have bond distances to the metal compatible with a direct coordination.^27^ An additional residue (Asp42 corresponding to Asp39 in *E. coli*) is spatially close and assumed by the authors to complete coordination.^9^ Arg124 (that is semiconserved as an Arg or Lys) is spatially close. A similar coordination sphere is observed in the structure from *T. thermophilus HB8* (pdb code: 2QQ4).

Zn^2+^ is also present in the PDB files of the NMR structures of *Haemophilus influenza* IscU (pdb codes: 1R9P and 1Q48) as it is directly circumstantiated by chemical shift analysis.^10^ Although Zn^2+^ cannot be detected by NMR directly, the authors provide convincing details in support of a role of His105 (which corresponds to His105 in *E. coli*) in coordination and modelled the zinc in the structure by imposing restraints that assume coordination by the three cysteines and the histidine. There is no mention about the Asp39 whose side chain points away from the direction where the ion would be hosted presumably because of lack of restraints. Interestingly, the authors report the presence of an exchange regime at this residue whose origin is, however, not discussed. This last observation prompted us to formulate the hypothesis that both His and Asp could have a role in coordination. Accordingly, we find that in the crystal structure of *E. coli* IscU complexed to IscS (pdb codes: 3LVL, 4EB5, and 4EB7), Asp39, His105 and the three cysteines point toward each other and are fully compatible with this hypothesis even though zinc is absent.^13^,^18^

### Copresence of Asp39 and His105 stabilizes *E. coli* IscU

**Figure 3:**
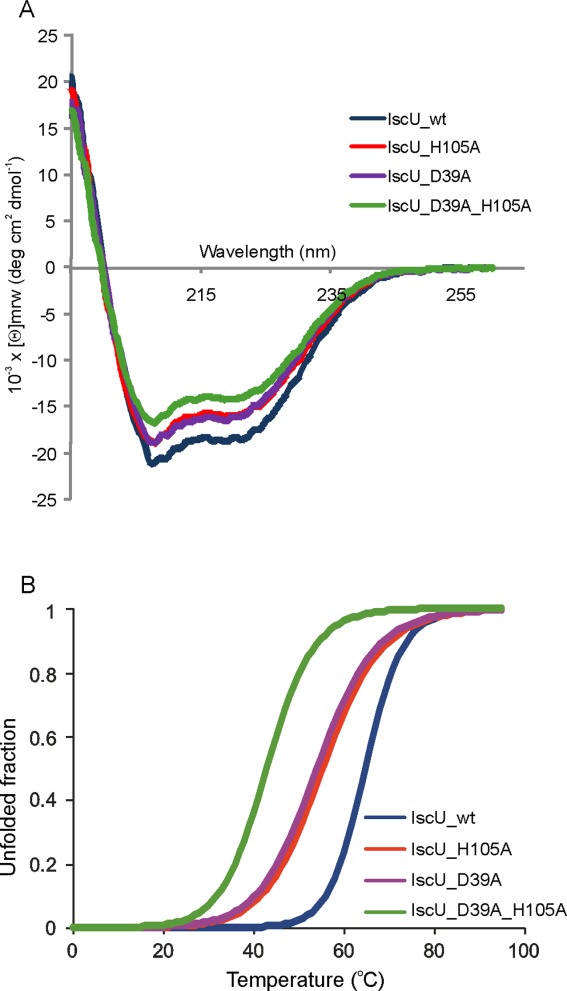
Comparison of the stabilities of IscU_wt with the IscU_D39A, IscU_H105A and IscU_D39A_H105A mutants. The proteins were as purified, that is, EDTA untreated. IscU_wt is indicated in black, IscU_D39A in purple, IscU_H105A in red and IscU_D39A_H105A in green. (A) Comparison of the far-UV spectra of the four proteins recorded at 25°C. (B) Thermal denaturation scans followed by detection of the CD signal at 222 nm.

To understand further the role of Asp39 and His105 in Zn^2+^ promoted stability, we compared experimentally the thermal unfolding curve of the wild-type protein with those of two single mutants (IscU_D39A and IscU_H105A) and of a double mutant (IscU_D39A_H105A). IscU_D39A has been extensively characterized and is known to stabilize the iron–sulfur cluster.^16^,^28^–^30^ The three mutants are correctly folded as demonstrated by their far-UV CD spectra (Fig. 3A) that are characterized by the two minima typical of predominantly helical proteins, but the molar ellipticities of the two single mutants are about 15% lower than that of IscU_wt, suggesting a lower content of secondary structure. An even lower content is observed for the double mutant.

Temperature scans carried out at pH 8 revealed that both the freshly purified single mutants have *T*_m_*s* about 10°C lower than IscU_wt **(**Fig. 3B and Table II**)**. This difference disappears after EDTA treatment indicating that the higher stability of IscU_wt reflects a higher affinity to zinc. Interestingly, the partially Zn^2+^-loaded single mutants have similar *T*_m_ values suggesting that they have similar affinities for the cation and capacity to retain the cation but lower than IscU_wt. The *T*_m_ of the double mutant does not change on EDTA pretreatment but increases considerably when adding Zn^2+^.

**Table II:** Comparison of the T_m_ Values (°C) of IscU With its Mutants at pH 8

	IscU_wt	IscU_D39A	IscU_H105A	IscU_D39A_H105A
As purified	64.8	53.7	55.4	43.1
Zn^2+^ free	42.0	43.7	41.2	43.0
Added Zn^2+^	68.2	67.1	66.3	65.4
Zn^2+^/Salt free	39.1	n.d.	n.d.	n.d.

These results indicate a role of both Asp39 and His105 in zinc coordination.

### Structural plasticity in Zn^2+^ coordination

To further understand Zn^2+^ coordination, we carried out quantum mechanics/molecular mechanics (QM/MM) calculations on IscU_wt and the single and double mutants. Although powerful, QM/MM calculations strongly depend on the choice of the starting conformation. To minimize the weight of the starting point, we used both a model of IscU built by homology of the crystallographic coordinates of 1SU0 and the X-ray structure of *E. coli* IscU in complex with IscS (3LVL) on which we added Zn^2+^. We did not detect appreciable differences between the results obtained from these two starting structures, and thus, present here the data obtained starting from 1SU0.

The p*K*_a_ of the Nɛ2 group of His105 was reported to be around 9, that is, at strong variance with the value reported for a random coil structure (around 6.8).^31^ The p*K*_a_ of the cysteines is usually ∼8 but we cannot exclude a more acidic behavior in the IscU environment as suggested in Ref.^22^ for SufU. Not to make assumptions on the protonation states, all combinations of protonated and deprotonated states of His105 and the cysteines were considered. We first optimized the geometries of the zinc free forms and then modeled Zn^2+^ in these. The initial and final structures superimpose with about 0.8 Å root mean square deviation (r.m.s.d.) on all the backbone atoms.

**Figure 4:**
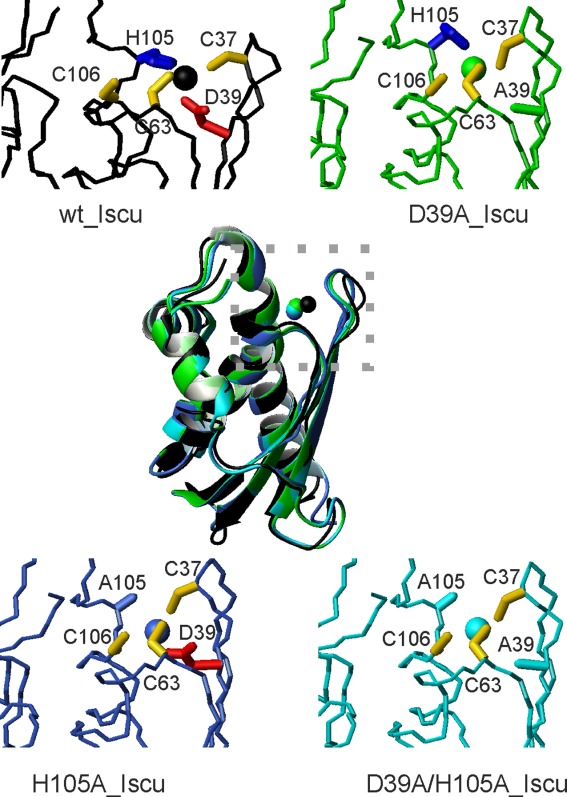
Final models of the zinc complexes of IscU_wt and its mutants after QM/MM calculation. In the center, is the structure superposition of the four structures assuming full deprotonation. Close ups on the region around the zinc-binding motif are shown for IscU_wt (black ribbon), IscU_D39A (green); IscU_H105A (blue), and IscU_D39A_H105A (cyan).

When the cysteines and His105 are all deprotonated, Zn^2+^ coordination of IscU_wt is approximately tetrahedral and mediated by Cys37, Cys63, Nɛ2 of His105 and the carboxylate group of Asp39 while Cys106 takes no part in coordination and points away from Zn^2+^ (Fig. 4). When the cysteines are deprotonated but His105 is protonated Cys106 participates to coordination while His105 is push out into the solvent (data not shown). In fully deprotonated IscU_D39A, Zn^2+^ is tetrahedrally coordinated by the three Cys thiol groups and by the deprotonated His105 side chain. In IscU_H105A, Zn^2+^ is coordinated by the three Cys and the Asp39 side chain (with one of the two oxygens being closer to Zn^2+^). In IscU_D39A_H105A, double mutant Zn^2+^ coordination is trigonal planar through the three Cys explaining why this mutant retains zinc less efficiently.^32^ The changes are directly reflected by the interatomic distances between the key residues of the zinc coordination shell (Supporting Information Tables S1 and S2). Mixed protonation states produce subtle changes of coordination, which again mainly involve rearrangements of the side chains.

**Figure 5:**
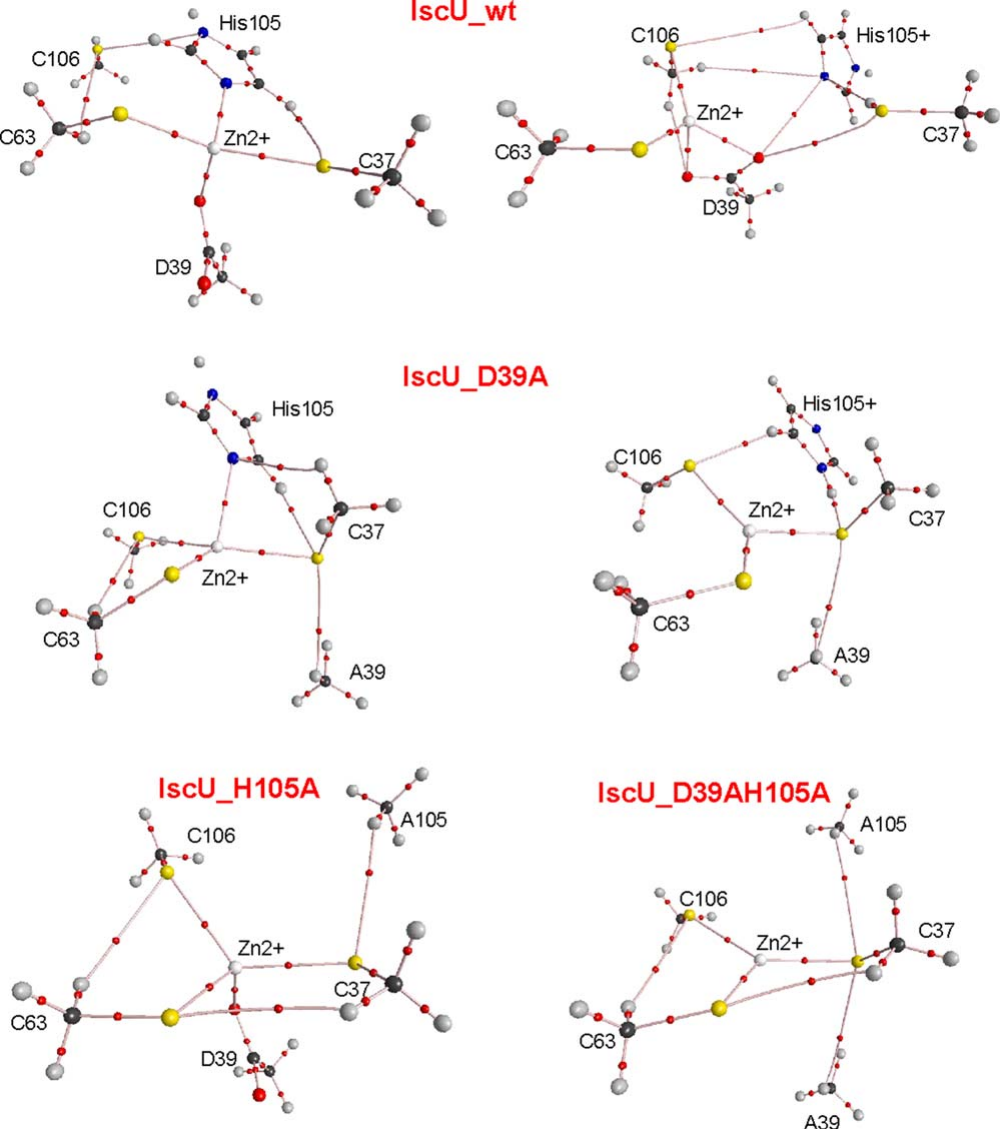
Location of the BCPs as respect to the atomic positions of the truncated models of IscU_wt and its mutants assuming deprotonated cysteines. Top two panels: His105 of IscU_wt is assumed uncharged (left) and charged (right); Middle panels: the same for IscU_D39A. Lower panel left: IscU_H105A. Lower panel right: IscU_D39A_H105A. BCPs are shown as small red spheres. Zn, S, O, C, H, and N atoms are shown as white, yellow, red, black, gray, and blue spheres, respectively.

The data were confirmed and extended by calculating the bond critical points (BCPs) and the presence of a bond path from the wave functions according the Bader's theory of “atoms in molecules” (AIM).^33^ According to this theory, identification of a BCP and the presence of a bond path between two atoms indicate a contact between atomic charge distributions, which can relate to the presence of an atomic bond between atoms or an electron density superposition. We observed values in the range 0.034–0.089 au for the pairs of atoms with distances below 3 Å (Supporting Information Table S3 and S4). These values are in the upper range determined for common hydrogen bonds (0.002–0.022 au) and approximately an order of magnitude smaller than those found for covalent bonds.^33^ The analysis fully supports a change of coordination for the different proteins according to their state of protonation. Visual inspection of BCPs also clarifies how coordination takes place (Fig. 5 and Supporting Information Figure S2). The states deprotonated on His105 are all consistent with a tetrahedral coordination. His105 protonation causes distortions from the tetrahedral geometry.

While it is difficult with this or other computational method to explore conformational states very distant from the starting points in the absence of experimental constraints, these results strongly support an extreme ductility of this system, which seems to have evolved to accommodate subtle variations of pH and sequence.

### Constitutive Zn^2+^ increases the efficiency of cluster formation

**Figure 6:**
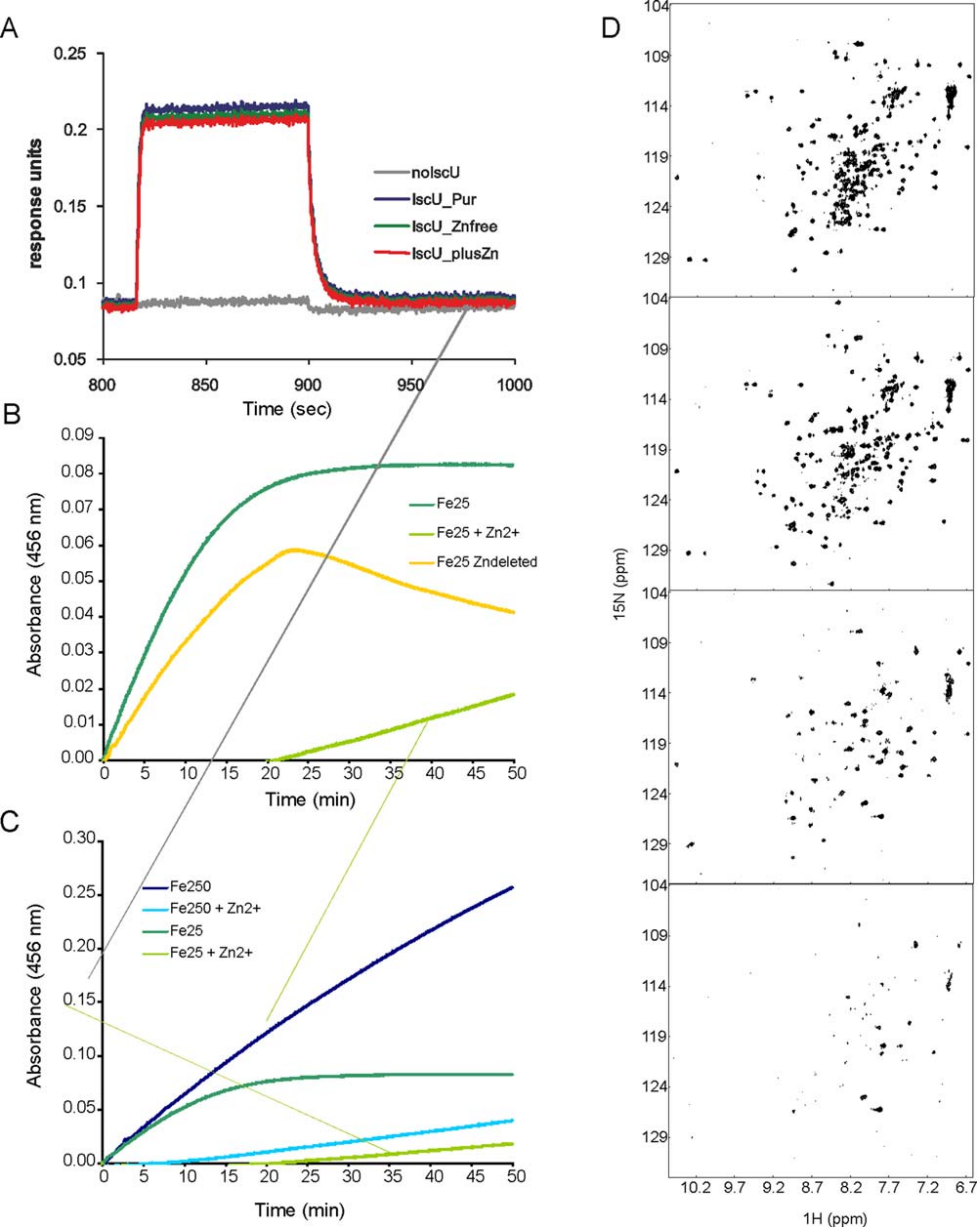
Effect of Zn^2+^ on IscU_wt properties. (A) BLI profile for the binding between immobilized IscS and different forms of IscU (as purified, Zn_free, Zn_saturated). The concentration of IscU used in this experiment was 1 µ*M* (below the Kd value) thus avoiding artifacts due to the saturation of IscS immobilized. (B) Kinetics of enzymatic cluster formation on IscU under strict anaerobic conditions using IscU (50 µ*M*), IscS (1 µ*M*), 250 µ*M* cysteine, 3 m*M* DTT, and 25 µ*M* Fe^2+^. IscU_wt was used as purified (dark green), as pretreated with EDTA (yellow), and as pretreated by EDTA to which Zn^2+^ was added (1:5; light green). (C) The same as in B but comparing the kinetics recorded using 25 µ*M* Fe^2+^ with 250 µ*M* Fe^2+^ and adding a 1:5 Zn^2+^ excess. (D) Effect of Zn^2+^ on the NMR HSQC spectrum of IscU_wt. From top to bottom: spectra of EDTA pretreated IscU_wt, titrated with 1:1, 5:1, and 10:1 Zn^2+^: protein molar equivalents. The spectra were recorded at 25°C and 600 MHz (Varian INOVA) in 10 m*M* in Tris–HCl buffer at pH 8.0.

To check whether zinc could interfere with protein–protein interactions, we used Biolayer interferometry (BLI)^34^ to compare the affinities of the complex with IscS in the presence and absence of Zn^2+^ using IscU as obtained from purification, treated by EDTA or Zn^2+^ saturated. No significant difference was observed in the binding of these three proteins: similar *K*_d_ values (1.5 ± 0.3 µ*M*) were obtained for the purified IscU, the Zn^2+^ free form and the Zn^2+^ saturated (Fig. 6A). These results suggest that the presence of Zn^2+^ on IscU does not appreciably influence IscS binding.

It was previously shown that the rate of cluster formation is reduced when Zn^2+^ is added (using 1:5 protein:Zn^2+^ molar ratios) to EDTA pretreated IscU.^20^ To check how the sample history might reflect on the enzymatic cluster formation, we recorded the IscS-mediated kinetics of cluster formation on IscU by absorbance (Fig. 6B). This *in vitro* assay is a well characterized and sensitive method to follow the enzymatic kinetics of IscS.^8^,^21^ We mixed under strict anaerobic conditions 50 µ*M* IscU, 1 µ*M* IscS, 250 µ*M* cysteine, 3 m*M* Dithiothreitol (DTT), and 25 µ*M* Fe^2+^ and followed the absorbance increase due to cluster formation. We used EDTA untreated IscU (as purified) and compared the kinetics of cluster formation with that obtained for the EDTA pretreated protein (1:500). We observed an appreciable increase of the kinetics and a higher efficiency for the sample as purified. This is the opposite of what independently reported in Refs.^20^ and^31^ but in excellent agreement with observations on SufU.^22^ We reasoned that our protein was EDTA untreated and thus contained Zn^2+^ or Zn^2+^ depleted by EDTA treatment, whereas the other authors compared a Zn^2+^-free sample with one posthumously treated with an excess (1:5) of zinc sulfate.

To understand whether this difference could account for the discrepancy, we repeated the experiment using conditions closer to the ones published by the other authors. We added a 1:5 protein:Zn^2+^ molar excess to EDTA pretreated IscU_wt. The presence of Zn^2+^considerably slowed down the reaction and leads to a global decrease of the overall signal, which has a lag time (Fig. 6B). As observed by these authors, the kinetics of EDTA pre-treated IscU_wt has a kink around 22 min, which we had never observed with our untreated samples,^8^,^21^ suggestive of the presence of two different phenomena.

To ensure that the difference is not determined by the much lower concentration of iron used in our assay as compared to Markley *et al*. (25 µ*M* vs. 250 µ*M*),^20^ we compared the effect of the Fe^2+^ concentration in the absence and in the presence of a 1:5 molar excess of Zn^2+^ (Fig. 6C). Also in this case, we observed the same relative behavior except for an overall increase of the absorbance as expected by increasing the Fe^2+^ substrate. The spectrum recorded in the presence of a Zn^2+^ excess retains a long lag phase.

Finally, to clarify the role of posthumously added zinc to the protein, we followed a zinc titration by NMR. We repeated the titration at low ionic strength and in the presence of 150 m*M* NaCl. In both cases, EDTA treatment (1:500 molar ratio) induces the presence of a second species, in addition to the stably folded one as it can be deduced by the appearance of extra resonances among which the splitting of the Trp side chain indole proton (Fig. 6D) as previously described.^20^ Addition of equimolar quantities of zinc seemed to clarify the spectrum stabilizing the folded species. However, higher molar ratios (1:5 and even more 1:10) of Zn^2+^ lead to clear protein aggregation and eventually to protein precipitation, which can be detected also visually. The effect is less dramatic when salt is present as no precipitation is observed but there is an overall broadening of the spectrum that is consistent with sample aggregation (Supporting Information Figure S3). These data also suggest that the stabilizing effect observed by CD when adding Zn^2+^ to Zn^2+^/salt-free IscU as compared to the *T*_m_ values observed for the as purified samples could reflect aggregation (Tables I and II).

Taken together, these r*e*sults tell us that posthumous addition of Zn^2+^interferes with cluster formation likely by promoting protein aggregation, whereas they point toward a beneficial effect of Zn^2+^ on the biological functions of IscU when the cation is constitutively present. Our data are in excellent agreement with observations on SufU.^22^

## Discussion

It has previously been reported that the fold of IscU is stabilized by zinc.^9^,^10^ It remains, however, unclear the *in vitro* and *in vivo* relevance of these findings. To gain further understanding on the matter, we investigated how Zn^2+^ binding affects the stability and the binding properties of IscU *in vitro*. The cation has such a strong affinity to the protein that remains bound even when IscU is overexpressed in its host organism. The Kd of the interaction between EDTA and zinc is 10^12^ *M*. An excess of 33 equivalents of EDTA was used to detach zinc from IscU. Based on the ratio IscU/EDTA used for metal chelation, this implies that the *K*_d_ of zinc binding can be estimated to be around 10^−13^ *M*. This is four order of magnitude lower than the value observed for SufU (10^−17^ *M*),^22^ which is among the highest binding constants reported for zinc-dependent enzymes.

Zinc has also a profound effect on the stability of the protein and causes a difference as large as 20°C on the melting temperatures of the Zn^2+^-loaded and Zn^2+^-free forms. In addition to a high temperature transition, we also observe a low temperature one, that is, indicative of cold denaturation.^25^ Depletion of Zn^2+^ is, however, insufficient to observe this second transition: it is necessary to additionally expose the protein to low ionic strengths. Interestingly, the stabilizing effect of ionic strength and specific divalent cations observed for IscU is strongly reminiscent of Yfh1, the yeast orthologue of frataxin. Like IscU, Yfh1 is a marginally stable acidic protein involved in iron–sulfur cluster biogenesis that at low ionic strengths undergoes cold and heat denaturation at detectable temperatures.^35^,^36^

Further, we questioned the role played by specific residues in stabilization. Both Asp39 and His105 are highly conserved residues and our results could explain why. We observe that, once depleted by ions, IscU_wt and its single mutants have comparable stabilities. At variance, when at least partially loaded by Zn^2+^, there is a consistent 10°C difference between wild-type and the single mutants indicating that both Asp39 and His105 can play a role in Zn^2+^ coordination (Table II). Accordingly, the as purified IscU_D39A_H105A mutant is approximately as unstable as the EDTA treated proteins.

Our calculations suggest that even modest rearrangements are sufficient to allow alternative zinc coordination showing large resilience and plasticity of the system. Depending on the protonation states of the various groups, zinc may be coordinated by alternative partners adopting different distorted tetrahedral geometries. This could provide a mechanism able to respond to even subtle changes of pH explaining the absolute conservation of the three cysteines while the aspartate and histidine can sometimes be alternatively substituted (usually by an alanine and a lysine, respectively). The hypothesis that the five residues are present to allow an octahedral coordination in which all three cysteines, Asp39 and His105 participate in coordination at the same time with the sixth ligand position probably occupied by a water molecule is in principle possible but does not at the moment find any experimental support.

Which consequences do our findings have for the biological function of IscU?

Because IscU an intracellular protein, it is highly unlikely that cold denaturation, which occurs in the absence of significant ionic strength, might have physiological relevance but our results bear important consequences for *in vitro* studies. Along the same line, it is interesting to observe that the *in vitro* results seem to be strongly dependent on sample history adding another layer of complexity to this already difficult-to-handle protein. Constitutively, incorporated zinc produces opposite results from those obtained after introducing zinc posthumously. This may not after all be too surprising as it is well-known that zinc induces protein aggregation: it is for instance known that zinc is the cation most commonly used for direct precipitation and as auxiliary agent.^37^ Zinc is also used in crystallization trials to induce precipitation. This is why, in the cell, total zinc is abundant (0.2 m*M*) but free zinc is only available in picomolar concentrations.^38^ Most of the cation is coordinated by transporters and released from them according to the Le Chatelier's law (zinc buffers).^38^

We did not observe significant variations of the affinity of IscU for IscS when the former is zinc loaded. While this is somewhat surprising as the stabilization effect of zinc could be expected to stabilize also this interaction, the effect could be below the sensitivity of BLI. What remains clear is that zinc facilitates rather than impeding iron–sulfur cluster formation when using samples with constitutively present zinc in agreement with what found for SufU.^22^ A higher rate of cluster formation in the presence of Zn^2+^ is consistent with a more stable fold and with the lower energetic cost of having a well preformed platform on which the cluster is assembled. These findings further indicate that the affinity of IscU for the cluster is much higher than that for zinc so that this ion can readily be expelled upon cluster formation. Finally, it is possible that zinc could act as a “redox zinc switch”^38^,^39^ by enhancing locally the effect of the reducing intracellular environment and preventing oxidation of the cysteines until the cluster is in place.

In conclusion, our results clarify several aspects of the behavior of IscU *in vitro* and support the hypothesis of a role of zinc in stabilizing the IscU fold and favor its function also *in vivo*. More work will need to be done to test this hypothesis further.

### Experimental Procedures

#### Protein purification

*E. coli* IscU_wt and its mutants were purified as previously described.^8^,^23^ In short, they were produced as fusion proteins with a His-tagged GST and purified by affinity chromatography using Ni-NTA agarose gel (QIAGEN). Cells overexpressing IscU were grown in Luria broth. Unless explicitly mentioned, we did not use the protocol previously described,^10^,^23^ which use enrichment of the medium with ZnSO_4_. All purification steps were carried out in the presence of 20 m*M* β-mercaptoethanol or 0.5 m*M* TCEP. The collected proteins were cleaved overnight from GST by TEV protease and further purified by gel-filtration chromatography on a Superdex 75 26/60 column (GE Healthcare). Protein purity was checked by SDS-PAGE and by mass spectrometry.

Unless otherwise specified, the Zn^2+-^free proteins were obtained treating the proteins with a 500 times excess of EDTA, which was then removed by gel filtration. When indicated, Zn^2+^ was added using a molar excess (1:3) of zinc sulfate to the purified IscU.

#### CD measurements

CD measurements were performed on a Jasco J-715 spectropolarimeter (Jasco UK, Great Dunmow, UK) equipped with a cell holder thermostatted by a PTC-348 Peltier system. Far-UV CD measurements were performed at 25°C in 20 m*M* phosphate buffer, 150 m*M* NaCl, 0.5 m*M* TCEP at pH values 6–9 using protein concentrations of 7–35 µ*M*. The spectra were recorded in fused silica cuvettes (Hellma) of 1 mm path length. Thermal unfolding was followed by monitoring the CD signal at 222 nm from 5 to 95°C at 1°C/min. The measurements were repeated at least three times.

Data were analyzed by direct nonlinear least-squares fitting of the observed far-UV CD signal (*Y*) to a two-state model of a single unfolding transition between native (N) and denatured (D) states:


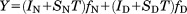


where *T* is the temperature (°C) and *I*_N_ (*I*_D_) and *S*_N_ (*S*_D_) are the intercepts and slopes for the optical signals of the native (denatured) forms. Free energy values used in calculating the fractional populations were obtained from the modified Gibbs–Helmholtz equation:^40^


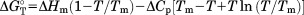


where *T*_m_ and Δ*H*_m_ are the temperature of the transition midpoint (K) and the van't Hoff enthalpy at *T*_m_ respectively, and Δ*C*_p_ is the difference in heat capacity between the native and denatured states.

#### NMR experiments

NMR HSQC experiments were carried out at 25°C and 600 MHz (Varian INOVA). Samples of ^15^N-labelled proteins (100–200 µ*M*) were prepared in buffer containing 5 m*M* Tris–HCl, 150 m*M* NaCl, 7% D_2_O, 2 m*M* TCEP, and 0.2 m*M* DSS.

#### Quantum mechanics calculations

We used as initial *E. coli* IscU_wt coordinates both chain A from the crystallographic structure 3LVL and a model built by homology using the crystal structure of IscU from *S. pyogenes* (1SU0) as a template (the two sets of coordinates differ by 0.8 Å r.m.s.d.). The mutants were built from both structures. The resulting models were prepared for QM/MM calculations by defining two layers. The QM region was defined around the cluster binding site and contained the side chains of the three conserved Cys, Asp39, and Ala39/Ala105, the imidazole ring of His105 and zinc (Supporting Information Figure S1). The other residues and the added water molecules were included in the MM layer.

Geometry optimizations of the IscU models were carried out using the two-layer ONIOM(B3LYP/GENECP:universal force field (UFF) = QEQ) method implemented in the Gaussian 09 program.^41^ The DFT-B3LYP approach^42^,^43^ was adopted for the QM layer and used in combination with the 6-311+G* basis set for H, C, N, S, and O atoms. The LANL2DZ effective core potential^44^ was used to represent the core electrons of the Zn^2+^ atom. The MM level was treated by the UFF with charges derived using the charge equilibration (QEQ) scheme.^45^ The r.m.s.d. of the *C*_α_ coordinates of each residue in the optimized holo proteins with respect to the input models are relatively small and similar for IscU_wt and the mutants with overall values of 1.4 and 1.2 Å, respectively.

Truncated models were built from the optimized QM/MM structures by considering only the atoms included in the QM layer (Supporting Information Figure S1). To satisfy the valence of the truncated carbon atoms, hydrogen atoms were added. The resulting structures were optimized at the B3LYP/6-311++G** theory level. During optimization, all heavy atoms were frozen at the corresponding positions obtained after the QM/MM optimization. Single point calculations were carried out on the resulting structures using the same theory level for all atoms and density-based solvation model (SMD) to simulate the effects of the polar solvent.^46^ The solvent assumed in the calculations was water with a dielectric constant *ɛ* of 78.4. Topological analysis of the computed wave functions at the SMD-B3LYP/6-311++G** level was performed using the AIM2000 package to quantify intra- and intermolecular interactions. Electron densities at the BCPs were evaluated.^47^,^48^ These parameters have widely been used to study intermolecular interactions and to demonstrate linear relations between bond stabilization energy and the increase in density at the BCP.

#### Biolayer Interferometry

All experiments were performed aerobically in 20 m*M* HEPES pH 7.5, 150 m*M* NaCl, 2 m*M* TCEP, 0.5 mg/mL BSA on an Octet Red instrument (ForteBio, Menlo Park, CA) operating at 25°C. Streptavidin-coated biosensors with immobilized biotinylated IscS were exposed to different concentrations of IscU (0–5 µ*M*).

#### Fe-S cluster reconstitution on IscU

Cluster reconstitution was performed under strict anaerobic conditions in a chamber (Belle technology) kept under nitrogen atmosphere. The reaction was followed by absorbance spectroscopy using a Cary 50 Bio spectrophotometer (Varian). Variations in the absorbance at 456 nm were measured as a function of time. A solution of 50 µ*M* IscU was incubated in sealed cuvettes typically using 3 m*M* DTT and 25 µ*M* Fe(NH_4_)_2_SO_4_ for 30 min in 50 m*M* Tris–HCl buffer, pH 7.5, and 150 m*M* NaCl. The reaction was initiated by adding IscS (1 µ*M*) and the reaction substrate cysteine (250 µ*M*). Each kinetic was repeated at least five times using independently purified protein batches.

## References

[1] Agar JN, Krebs C, Frazzon J, Huynh BH, Dean DR, Johnson MK (2000). IscU as a scaffold for iron-sulfur cluster biosynthesis: sequential assembly of 2Fe-2S and 4Fe-4S clusters in IscU. Biochemistry.

[2] Garland SA, Hoff K, Vickery LE, Culotta VC (1999). *Saccharomyces cerevisiae* ISU1 and ISU2: members of a well-conserved gene family for iron-sulfur cluster assembly. J Mol Biol.

[3] Hwang DM, Dempsey A, Tan KT, Liew CC (1996). A modular domain of NifU, a nitrogen fixation cluster protein, is highly conserved in evolution. J Mol Evol.

[4] Zheng LM, Cash VL, Flint DH, Dean DR (1998). Assembly of iron-sulfur clusters—identification of an iscSUA-hscBA-fdx gene cluster from *Azotobacter vinelandii*. J Biol Chem.

[5] Lill R, Hoffmann B, Molik S, Pierik AJ, Rietzschel N, Stehling O, Uzarska MA, Webert H, Wilbrecht C, Muehlenhoff U (2012). The role of mitochondria in cellular iron-sulfur protein biogenesis and iron metabolism. Biochim Biophys Acta.

[6] Roche B, Aussel L, Ezraty B, Mandin P, Py B, Barras F (2013). Iron/sulfur proteins biogenesis in prokaryotes: formation, regulation and diversity. Biochim Biophys Acta.

[7] Kato S, Mihara H, Kurihara T, Takahashi Y, Tokumoto U, Yoshimura T, Esaki N (2002). Cys-328 of IscS and Cys-63 of IscU are the sites of disulfide bridge formation in a covalently bound IscS/IscU complex: implications for the mechanism of iron-sulfur cluster assembly. Proc Natl Acad Sci USA.

[8] Adinolfi S, Iannuzzi C, Prischi F, Pastore C, Iametti S, Martin SR, Bonomi F, Pastore A (2009). Bacterial frataxin CyaY is the gatekeeper of iron-sulfur cluster formation catalyzed by IscS. Nat Struct Mol Biol.

[9] Liu JY, Oganesyan N, Shin DH, Jancarik J, Yokota H, Kim R, Kim SH (2005). Structural characterization of an iron-sulfur cluster assembly protein IscU in a zinc-bound form. Proteins.

[10] Ramelot TA, Cort JR, Goldsmith-Fischman S, Kornhaber GJ, Xiao R, Shastry R, Acton TB, Honig B, Montelione GT, Kennedy MA (2004). Solution NMR structure of the iron-sulfur cluster assembly protein U (IscU) with zinc bound at the active site. J Mol Biol.

[11] Shimomura Y, Wada K, Fukuyama K, Takahashi Y (2008). The asymmetric trimeric architecture of 2Fe-2S IscU: implications for its scaffolding during iron-sulfur cluster biosynthesis. J Mol Biol.

[12] Johnson DC, Dean DR, Smith AD, Johnson MK (2005). Structure, function, and formation of biological iron-sulfur clusters. Annu Rev Biochem.

[13] Marinoni EN, de Oliveira JS, Nicolet Y, Raulfs EC, Amara P, Dean DR, Fontecilla-Camps JC (2012). (IscS-IscU)2 complex structures provide insights into Fe2S2 biogenesis and transfer. Angew Chem Int Ed.

[14] Shi R, Proteau A, Villarroya M, Moukadiri I, Zhang LH, Trempe JF, Matte A, Armengod ME, Cygler M (2010). Structural basis for Fe-S cluster assembly and tRNA thiolation mediated by IscS protein-protein interactions. PLoS Biol.

[15] Yamanak Y, Zeppieri L, Nicolet Y, Marinoni EN, De Oliveira JS, Odaka M, Dean DR, Fontecilla-Camps JC (2013). Crystal structure and functional studies of an unusual L-cysteine desulfurase from *Archaeoglobus fulgidus*. Dalton Trans.

[16] Unciuleac M-C, Chandramouli K, Naik S, Mayer S, Huynh BH, Johnson MK, Dean DR (2007). In vitro activation of apo-aconitase using a [4Fe-4S] cluster-loaded form of the IscU [Fe-S] cluster scaffolding protein. Biochemistry.

[17] Bonomi F, Iametti S, Morleo A, Ta D, Vickery LE (2011). Facilitated transfer of IscU-[2Fe2S] clusters by chaperone-mediated ligand exchange. Biochemistry.

[18] Riboldi GP, Verli H, Frazzon J (2009). Structural studies of the *Enterococcus faecalis* SufU [Fe-S] cluster protein. BMC Biochem.

[19] Kornhaber GJ, Snyder D, Moseley HNB, Montelione GT (2006). Identification of zinc-ligated cysteine residues based on C-13 alpha and C-13 beta chemical shift data. J Biomol NMR.

[20] Markley JL, Kim JH, Dai Z, Bothe JR, Cai K, Frederick RO, Tonelli M (2013). Metamorphic protein IscU alternates conformations in the course of its role as the scaffold protein for iron-sulfur cluster biosynthesis and delivery. FEBS Lett.

[21] Prischi F, Konarev PV, Iannuzzi C, Pastore C, Adinolfi S, Martin SR, Svergun DI, Pastore A (2010). Structural bases for the interaction of frataxin with the central components of iron-sulphur cluster assembly. Nat Commun.

[22] Selbach BP, Chung AH, Scott AD, George SJ, Cramer SP, Dos Santos PC (2014). Fe-S cluster biogenesis in gram-positive bacteria: SufU is a zinc-dependent sulfur transfer protein. Biochemistry.

[23] Prischi F, Pastore C, Carroni M, Iannuzzi C, Adinolfi S, Temussi P, Pastore A (2010). Of the vulnerability of orphan complex proteins: the case study of the *E. coli* IscU and IscS proteins. Protein Expr Purif.

[24] Adinolfi S, Rizzo F, Masino L, Nair M, Martin SR, Pastore A, Temussi PA (2004). Bacterial IscU is a well folded and functional single domain protein. Eur J Biochem.

[25] Privalov PL (1990). Cold denaturation of proteins. Crit Rev Biochem Mol Biol.

[26] Myers JK, Pace CN, Scholtz JM (1995). Denaturant M-values and heat-capacity changes—relation to changes in accessible surface-areas of protein unfolding. Protein Sci.

[27] Harding MM (2004). The architecture of metal coordination groups in proteins. Acta Cryst.

[28] Foster MW, Mansy SS, Hwang J, Penner-Hahn JE, Surerus KK, Cowan JA (2000). A mutant human IscU protein contains a stable [2Fe-2S](2+) center of possible functional significance. J Am Chem Soc.

[29] Wu G, Mansy SS, Hemann C, Hille R, Surerus KK, Cowan JA (2002). Iron-sulfur cluster biosynthesis: characterization of *Schizosaccharomyces pombe* Isa1. J Biol Inorg Chem.

[30] Wu SP, Wu G, Surerus KK, Cowan JA (2002). Iron-sulfur cluster biosynthesis. Kinetic analysis of [2Fe-2S] cluster transfer from holo ISU to apo Fd: role of redox chemistry and a conserved aspartate. Biochemistry.

[31] Dai Z, Tonelli M, Markley JL (2012). Metamorphic protein IscU changes conformation by cis-trans isomerizations of two peptidyl-prolyl peptide bonds. Biochemistry.

[32] Gruff ES, Koch SA (1989). A trigonal planar [Zn(Sr)3]1-complex—a possible new coordination mode for zinc cysteine centers. J Am Chem Soc.

[33] Bader RF (1990). Atoms and Molecules: A Quantum Theory.

[34] Concepcion J, K Witte, C Wartchow, S Choo, D Yao, H Persson, J Wei, P Li, B Heidecker, W Ma, R Varma, LS Zhao, D Perillat, G Carricato, M Recknor, K Du, H Ho, T Ellis, J Gamez, M Howes, J Phi-Wilson, S Lockard, R Zuk, H Tan (2009). Label-free detection of biomolecular interactions using biolayer interferometry for kinetic characterization. Comb Chem High Throughput Screen.

[35] Adrover M, Esposito V, Martorell G, Pastore A, Temussi PA (2010). Understanding cold denaturation: the case study of Yfh1. J Am Chem Soc.

[36] Pastore A, Martin SR, Politou A, Kondapalli KC, Stemmler T, Temussi PA (2007). Unbiased cold denaturation: low- and high-temperature unfolding of yeast frataxin under physiological conditions. J Am Chem Soc.

[37] Cohn EJ, Gurd FRN, Surgenor DM, Barnes BA, Brown RK, Derouaux G, Gillespie JM, Kahnt FW, Lever WF, Liu CH, Mittelman D, Mouton RF, Schmid K, Uroma E (1950). A system for the separation of the components of human blood: quantitative procedures for the separation of the protein components of human plasma. J Am Chem Soc.

[38] Maret W (2006). Zinc coordination environments in proteins as redox sensors and signal transducers. Antioxidants Redox Signal.

[39] Morgan B, Ang SK, Yan G, Lu H (2009). Zinc can play chaperone-like and inhibitor roles during import of mitochondrial small Tim proteins. J Biol Chem.

[40] Becktel WJ, Schellman JA (1987). Protein stability curves. Biopolymers.

[41] Frisch MJ, GW Trucks, HB Schlegel, GE Scuseria, MA Robb, JR Cheeseman, G Scalmani, V Barone, B Mennucci, GA Petersson, H Nakatsuji, M Caricato, X Li, HP Hratchian, AF Izmaylov, J Bloino, G Zheng, JL Sonnenberg, M Hada, M Ehara, K Toyota, R Fukuda, J Hasegawa, M Ishida, T Nakajima, Y Honda, O Kitao, H Nakai, T Vreven, JA MontgomeryJr, JE Peralta, F Ogliaro, M Bearpark, JJ Heyd, E Brothers, KN Kudin, N StaroverovV, T Keith, R Kobayashi, J Normand, K Raghavachari, A Rendell, JC Burant, SS Iyengar, J Tomasi, M Cossi, N Rega, JM Millam, M Klene, JE Knox, JB Cross, V Bakken, C Adamo, J Jaramillo, R Gomperts, RE Stratmann, O Yazyev, AJ Austin, R Cammi, C Pomelli, JW Ochterski, RL Martin, K Morokuma, VG Zakrzewski, GA Voth, P Salvador, JJ Dannenberg, S Dapprich, AD Daniels, O Farkas, JB Foresman, JV Ortiz, J Cioslowski, DJ Fox

[42] Becke AD (1993). A new mixing of Hartree-Fock and local density-functional theories. J Chem Phys.

[43] Lee CT, Yang WT, Parr RG (1988). Development of the Colle-Salvetti correlation-energy formula into a functional of the electron-density. Phys Rev B.

[44] Hay PJ, Wadt WR (1985). Ab initio effective core potentials for molecular calculations—potentials for the transition-metal atoms Sc to Hg. J Chem Phys.

[45] Rappe AK, Goddard WA (1991). Charge equilibration for molecular-dynamics simulations. J Phys Chem.

[46] Marenich AV, Cramer CJ, Truhlar DG (2009). Universal solvation model based on solute electron density and on a continuum model of the solvent defined by the bulk dielectric constant and atomic surface tensions. J Phys Chem B.

[47] Grabowski SJ (2001). A new measure of hydrogen bonding strength—ab initio and atoms in molecules studies. Chem Phys Lett.

[48] LaPointe SM, Farrag S, Bohorquez HJ, Boyd RJ (2009). QTAIM study of an alpha-helix hydrogen bond network. J Phys Chem B.

