# Supplementary material for: The role of zinc in the stability of the marginally stable IscU scaffold protein
Source: Protein Sci. 2014 Jun 11;23(9):1208–19. doi: 10.1002/pro.2501 (PMC4243993; doi:10.1002/pro.2501)
Supplement: Supplementary file 1 — Supplementary Information [file pro0023-1208-sd1.pdf]

**Suppl. Materials**

**Figure S1 -** System preparation. Truncated models of holo IscU_wt used in DFT calculations. Hydrogen atoms were added to compensate the valence of the truncated atoms.

**Figure S2** - Location of the BCPs as respect to the atomic positions of the truncated models of IscU_wt and its mutants as in Figure 5 but assuming protonated cysteines. Top two panels: His105 of IscU_wt is assumed uncharged (left) and charged (right); Middle panels: the same for IscU_D39A. Lower panel left: IscU_H105A. Lower panel right: IscU_D39A_H105A. BCPs are shown as small red spheres. Zn, S, O, C, H, and N atoms are shown as white, yellow, red, black, grey, and blue spheres respectively.

**Figure S3 –** Effect of $Zn^{2+}$ on the NMR HSQC spectrum of IscU_wt as in Figure 6D but in the presence of 150 mM NaCl. From top to bottom: Spectra of EDTA pre-treated IscU_wt, titrated with 1:1 and 5:1 $Zn^{2+}$: protein molar equivalents.

**Table S1.** Geometric parameters of the coordination site in zinc loaded IscU with all Cys protonated. The distances were taken from the structures optimized at the detailed QM/MM computational level. Numbering is according to Figure S1.

| | Wt ($H105^+$) | Wt ($H105^0$) | D39A[a] ($H105^+$) | D39A[a] ($H105^0$) | H105A | D39A_H105A |
|---|---|---|---|---|---|---|
| **Distances (Å)** | | | | | | |
| Zn-S(5) | 2.604 | 4.252 | 2.484 | 2.633 | 2.974 | 2.495 |
| Zn-S(7) | 2.554 | 2.493 | 2.468 | 2.560 | 2.509 | 2.521 |
| Zn-O(12) | 2.092 | 2.045 | -- | -- | 2.023 | -- |
| Zn-O(13) | 2.108 | 2.113 | -- | -- | 2.203 | -- |
| Zn-S(10) | 2.746 | 4.081 | 2.467 | 2.582 | 2.580 | 2.489 |
| Zn-N(9) | 8.184 | 2.065 | 9.293 | 2.054 | -- | -- |
| SH(5)-O(12) | 4.488 | 2.954 | -- | -- | 2.459 | -- |
| SH(10)-O(13) | 2.608 | 2.348 | -- | -- | 2.269 | -- |
| **Angles (*deg*)** | | | | | | |
| O(12)-HS(5)-S(5) | 42.6 | 151.0 | -- | -- | 118.6 | -- |
| O(13)-HS(10)-S(10) | 120.4 | 143.8 | -- | -- | 121.4 | -- |
| O(13)-HS(7)-S(7) | 33.1 | 143.8 | -- | -- | -- | -- |
| S(7)-Zn-N(7) | 144.1 | 110.6 | -- | 136.15 | -- | -- |
| O(12)-Zn-O(13) | 62.73 | 63.6 | -- | -- | 62.2 | -- |
| **Dihedral Angles (*deg*)** | | | | | | |
| S(7)-N(9)-O(13)-O(12) | 87.1 | 84.9 | -- | -- | -- | -- |
| S(5)-S(7)-Zn-S(10) | 116.9 | -- | 150.9 | -- | -- | 156.19 |
| S(7)-N(9)-S(10)-S(5) | -5.6 | -- | -- | 51.5 | -- | -- |
| S(7)-S(10)-O(13)-O(12) | 117.9 | -- | -- | -- | 88.2 | -- |

**Table S2.** Geometric parameters of the coordination site in zinc loaded IscU when all Cys are deprotonated. The distances were taken from the structures optimized at the detailed QM/MM computational level. Numbering is according to Figure S1.

| | Wt ($H105^+$) | Wt ($H105^0$) | D39A[a] ($H105^+$) | D39A[a] ($H105^0$) | H105A | D39A_H105A |
|---|---|---|---|---|---|---|
| **Distances (Å)** | | | | | | |
| Zn-S(5) | 2.396 | 5.706 | 2.389 | 2.518 | 2.585 | 2.419 |
| Zn-S(7) | 2.372 | 2.392 | 2.319 | 2.419 | 2.484 | 2.391 |
| Zn-O(12) | 2.202 | 2.050 | -- | -- | 2.095 | -- |
| Zn-O(13) | 2.241 | 2.997 | -- | -- | 2.982 | -- |
| Zn-S(10) | 4.826 | 2.414 | 2.479 | 2.485 | 2.541 | 2.405 |
| Zn-N(9) | 7.623 | 2.211 | 4.422 | 2.351 | -- | -- |
| **Angles (*deg*)** | | | | | | |
| S(5)-Zn-S(7) | 121.2 | 73.3 | 126.2 | 101.1 | 96.3 | 107.1 |
| N(9)-Zn-O(12) | 81.9 | 93.5 | -- | -- | -- | -- |
| N(9)-Zn-S(10) | 47.0 | 92.2 | 44.0 | 86.06 | -- | -- |
| S(7)-Zn-N(9) | 146.7 | 105.4 | 62.8 | 117.5 | -- | -- |
| O(12)-Zn-O(13) | 59.6 | 48.1 | -- | -- | 48.6 | -- |
| **Dihedral Angles (*deg*)** | | | | | | |
| S(7)-N(9)-O(13)-O(12) | 102.1 | 120.5 | -- | -- | -- | -- |
| S(5)-S(7)-Zn-S(10) | 115.4 | 146.5 | 153.6 | -171.9 | 130.5 | 165.6 |
| S(7)-Zn-N(9)-S(10) | 10.0 | 141.8 | 34.2 | 111.5 | -- | -- |
| N(9)-Zn-O(12)-O(13) | 41.3 | 168.8 | -- | -- | -- | -- |

**Table S3.** Electron density at the bond critical points [$\rho(r_c)$ (au)] of IscU as calculated assuming protonated cysteines. BCP were derived from the wave function calculated at the SMD-B3LYP/6-311++G** computational level.

| | BCP | | | | | |
|---|---|---|---|---|---|---|
| | Wt ($H105^+$) | Wt ($H105^0$) | D39A[a] ($H105^+$) | D39A[a] ($H105^0$) | H105A | D39A_H105A |
| **Zn-S(5)** | 0.041 | -- | 0.051 | 0.035 | 0.017 | 0.049 |
| **Zn-S(7)** | 0.046 | 0.050 | 0.055 | 0.045 | 0.050 | 0.049 |
| **Zn-O(12)** | 0.064 | 0.069 | -- | -- | 0.073 | -- |
| **Zn-O(13)** | 0.061 | 0.058 | -- | -- | 0.049 | -- |
| **Zn-N(9)** | -- | 0.074 | -- | 0.076 | -- | -- |
| **Zn-S(10)** | 0.030 | -- | 0.055 | 0.044 | 0.044 | 0.053 |
| **SH(5)-O(12)** | -- | 0.003 | -- | -- | 0.010 | -- |
| **SH(10)-O(13)** | -- | 0.011 | -- | -- | 0.016 | -- |

**Table S4.** Electron density at the bond critical points [$\rho(r_c)$ (au)] of IscU as calculated assuming deprotonated cysteines. BCP were derived from the wave function calculated at the SMD-B3LYP/6-311++G** computational level.

| | BCP | | | | | |
|---|---|---|---|---|---|---|
| | Wt ($H105^+$) | Wt ($H105^0$) | D39A[a] ($H105^+$) | D39A[a] ($H105^0$) | H105A | D39A_H105A |
| **Zn-S(5)** | 0.062 | -- | 0.061 | 0.046 | 0.042 | 0.056 |
| **Zn-S(7)** | 0.065 | 0.063 | 0.071 | 0.059 | 0.053 | 0.062 |
| **Zn-O(12)** | 0.049 | 0.067 | -- | -- | 0.061 | -- |
| **Zn-O(13)** | 0.045 | -- | -- | -- | -- | -- |
| **Zn-N(9)** | -- | 0.054 | -- | 0.040 | -- | -- |
| **Zn-S(10)** | -- | 0.058 | 0.054 | 0.054 | 0.048 | 0.062 |
| **S(10)-O(13)** | 0.004 | -- | -- | -- | -- | -- |
| **S(10)-$NH^+$(9)** | -- | -- | 0.053 | -- | -- | -- |

Figure S1

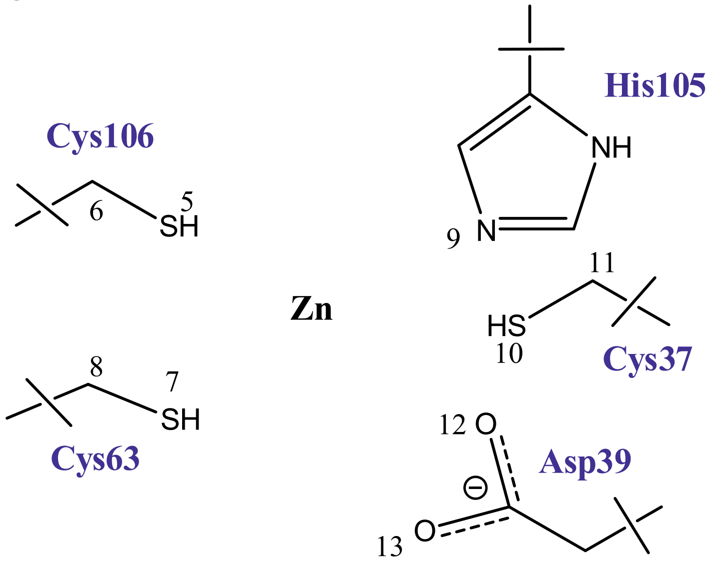

His105
NH
9 N
Cys106
6
5
SH
Zn
11
HS
10
Cys37
8
7
SH
Cys63
12 O
Asp39
13 O

# Figure S2

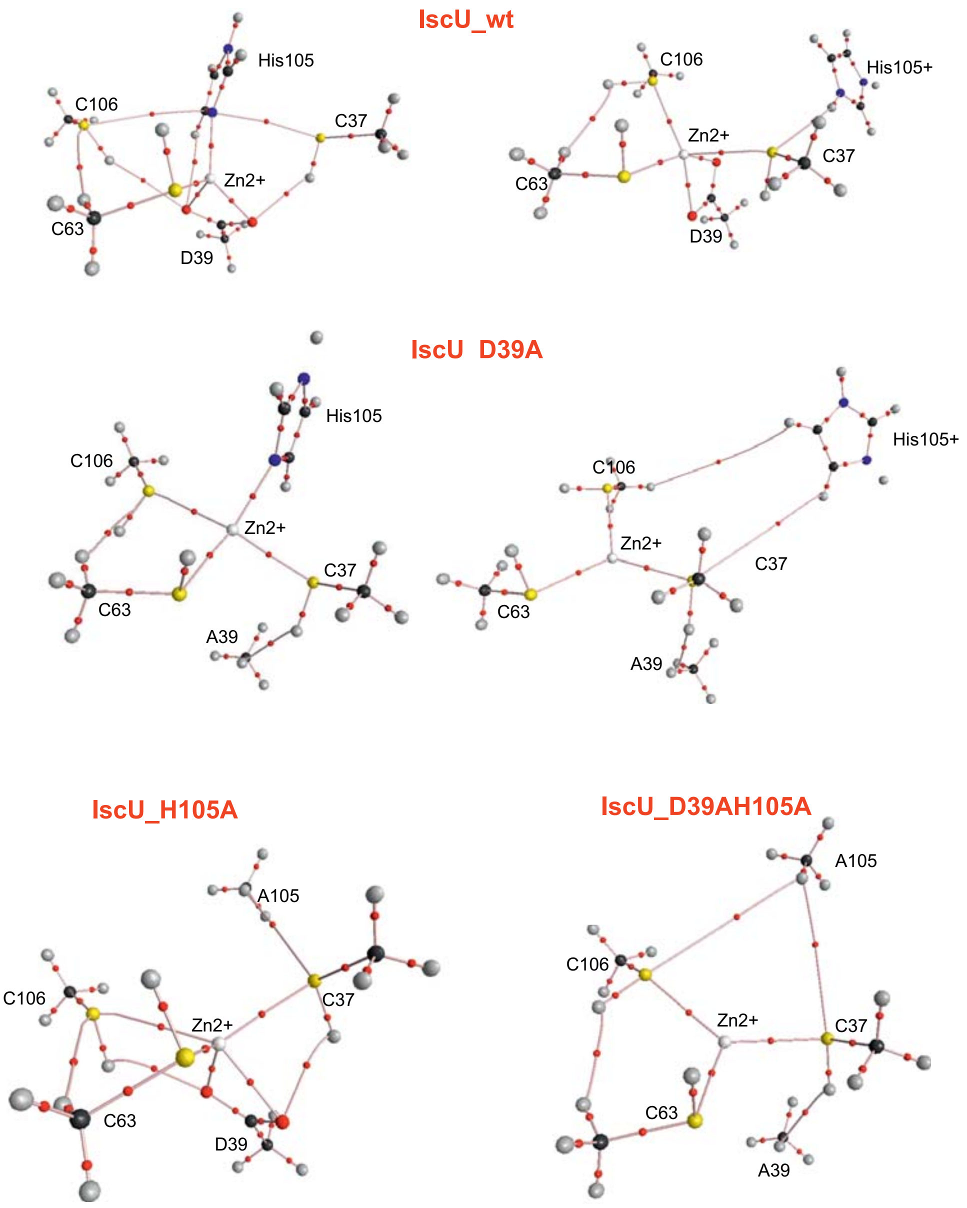
IscU_wt
His105
C106
C37
Zn2+
C63
D39
C106
His105+
Zn2+
C37
C63
D39
IscU D39A
His105
C106
Zn2+
C37
C63
A39
His105+
C106
Zn2+
C37
C63
A39
IscU_H105A
A105
C106
Zn2+
C37
C63
D39
IscU_D39AH105A
A105
C106
Zn2+
C37
C63
A39

# Figure S3

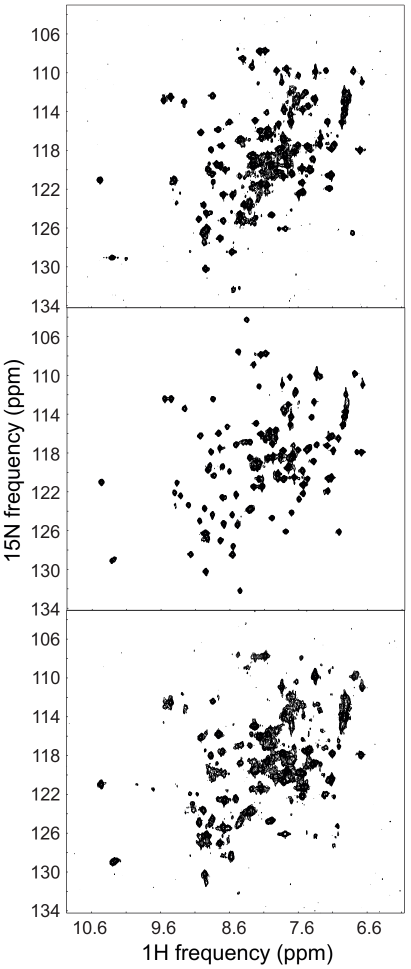

106
110
114
118
122
126
130
134
15N frequency (ppm)
10.6
9.6
8.6
7.6
6.6
1H frequency (ppm)
